# Association between eGDR and MASLD and liver fibrosis: a cross-sectional study based on NHANES 2017–2023

**DOI:** 10.3389/fmed.2025.1579879

**Published:** 2025-05-30

**Authors:** Wenjing Peng, Zeyu Li, Nian Fu

**Affiliations:** ^1^The Affiliated Nanhua Hospital, Department of Gastroenterology, Clinical Research Center for Metabolic Associated Fatty Liver Disease in Hunan Province, Hengyang Medical School, University of South China, Hengyang, China; ^2^Department of Thyroid Surgery, The Second Xiangya Hospital, Central South University, Changsha, China

**Keywords:** eGDR, MASLD, insulin resistance, diabetes, NHANES, cross-sectional study

## Abstract

**Background:**

This study aimed to investigate the association between estimated glucose disposal rate (eGDR) and metabolic dysfunction-associated steatotic liver disease (MASLD), as well as liver fibrosis, using data from the National Health and Nutrition Examination Survey (NHANES) 2017–2023 dataset.

**Methods:**

Data from 7,855 participants in the NHANES 2017–2023 dataset were analyzed. Multivariable logistic regression models were constructed to assess the association between eGDR (both continuous and quartiles) and MASLD, as well as liver fibrosis, adjusting for potential confounders. Generalized additive models (GAM) were used to explore non-linear relationships, stratified by age, hypertension, diabetes, cardiovascular disease (CVD), and body mass index (BMI). A two-piecewise linear regression model was used to examine threshold effects. Subgroup analyses were conducted to assess effect modification. Mediation analysis was performed to determine the role of the atherogenic index of plasma (AIP). Sensitivity analysis was performed to test the robustness of the results.

**Results:**

In the fully adjusted model, higher eGDR was inversely associated with both MASLD and liver fibrosis (MASLD: OR = 0.62, 95% CI: 0.53–0.72, *p* < 0.0001; liver fibrosis: OR = 0.50, 95% CI: 0.42–0.58, *p* < 0.0001). Participants in higher eGDR quartiles (Q2, Q3, and Q4) had progressively lower odds of both MASLD and liver fibrosis compared to those in Q1 (MASLD: Q2: OR = 0.56, 95% CI: 0.37–0.84, *p* = 0.0047; Q3: OR = 0.25, 95% CI: 0.12–0.50, *p* = 0.0001; Q4: OR = 0.13, 95% CI: 0.05–0.31, *p* < 0.0001; liver fibrosis: Q2: OR = 0.24, 95% CI: 0.13–0.44, *p* < 0.0001; Q3: OR = 0.06, 95% CI: 0.02–0.16, *p* < 0.0001; Q4: OR = 0.05, 95% CI: 0.01–0.19, *p* < 0.0001). A non-linear relationship with threshold effects at an eGDR value of 3.25 was observed for MASLD. Subgroup analyses revealed that the inverse association between eGDR and MASLD was more pronounced in individuals without diabetes. Additionally, smoothing curve fitting showed that the dose–response relationship between eGDR and both MASLD and liver fibrosis differed by metabolic and clinical status. Mediation analysis suggested that AIP partially mediated the association between eGDR and MASLD, accounting for approximately 10.6% of the total effect. Sensitivity analyses excluding extreme eGDR values confirmed the robust inverse associations with MASLD and liver fibrosis.

**Conclusion:**

This study found a significant non-linear inverse association between eGDR and both MASLD and liver fibrosis, with a threshold effect observed for MASLD. The association was stronger in non-diabetic individuals and partially mediated by AIP. Moreover, the dose–response relationships varied across metabolic and clinical subgroups.

## 1 Introduction

Metabolic dysfunction-associated steatotic liver disease (MASLD), formerly known as nonalcoholic fatty liver disease (NAFLD), is a prevalent chronic liver condition characterized by hepatic steatosis in individuals with metabolic dysfunction (1). Affecting an estimated 30% of the global population, approximately 1.6 billion individuals, MASLD is recognized as a significant and growing public health concern (2). The prevalence varies geographically, with higher rates observed in Latin America, the Middle East, and South Asia (3). While genetic factors contribute to the risk of MASLD (4), racial, ethnic, and socioeconomic disparities also play a crucial role in disease incidence and severity (5, 6). The presence of hepatic fibrosis is a critical determinant of liver-related morbidity and mortality, with advanced fibrosis stages associated with increased risk of liver failure and hepatocellular carcinoma (7). Furthermore, MASLD is strongly linked to metabolic syndrome and its associated comorbidities, including type 2 diabetes, cardiovascular disease (CVD), and chronic kidney disease (8, 9). Even individuals with MASLD-related cirrhosis are more likely to die from cardiovascular events than liver-related causes (10). Given the high global prevalence, potential for severe complications, and association with other systemic diseases, MASLD poses a substantial burden on healthcare systems worldwide.

Insulin resistance (IR) is a critical factor in the development and progression of MASLD (11). Defined as a reduced efficiency of insulin in promoting glucose uptake and utilization (12), IR is a key component of metabolic syndrome and is closely associated with a range of metabolic disorders (13, 14). While the hyperinsulinemic-euglycemic clamp is the gold standard for quantifying insulin sensitivity, its invasiveness and high-cost limit its use in large-scale clinical studies (15, 16). As a result, surrogate markers of IR, such as the homeostatic model assessment of insulin resistance and the triglyceride-glucose (TyG) index, have been developed (17, 18). However, these measures may be influenced by insulin use or provide less accurate assessments in certain populations (19).

The estimated glucose disposal rate (eGDR), initially developed to assess IR in type 1 diabetes, has emerged as a promising alternative (20). Studies suggest eGDR offers improved accuracy compared to the euglycemic-hyperinsulinemic clamp and is suitable for large-scale clinical research (21). Furthermore, lower eGDR has been associated with increased risk and adverse outcomes in various conditions, including acute coronary syndrome (22), heart failure (23) and stroke (21). Previous small-sample studies have suggested a significant association between eGDR and NAFLD in individuals with type 1 diabetes (19, 24). In 2024, Song et al. (25) conducted a cross-sectional study and found that eGDR was inversely associated with all-cause and cardiovascular mortality in NAFLD. Takahiro et al. (26) conducted a longitudinal cohort study in Japan, which recruited 16,689 participants, and found that lower eGDR levels were associated with an increased risk of MASLD. However, research on eGDR in the context of MASLD remains limited. To address this gap, our study leverages data from the National Health and Nutrition Examination Survey (NHANES) to investigate the relationship between eGDR and both MASLD and liver fibrosis in a large, nationally representative sample. This analysis will stratify the population to examine the predictive capacity of eGDR across diverse subgroups, offering insights for targeted interventions. Furthermore, we will explore the mediating role of the atherogenic index of plasma (AIP) in the relationship between eGDR and MASLD, providing a more comprehensive understanding of the underlying mechanisms.

## 2 Materials and methods

### 2.1 Study population

**Figure 1:**
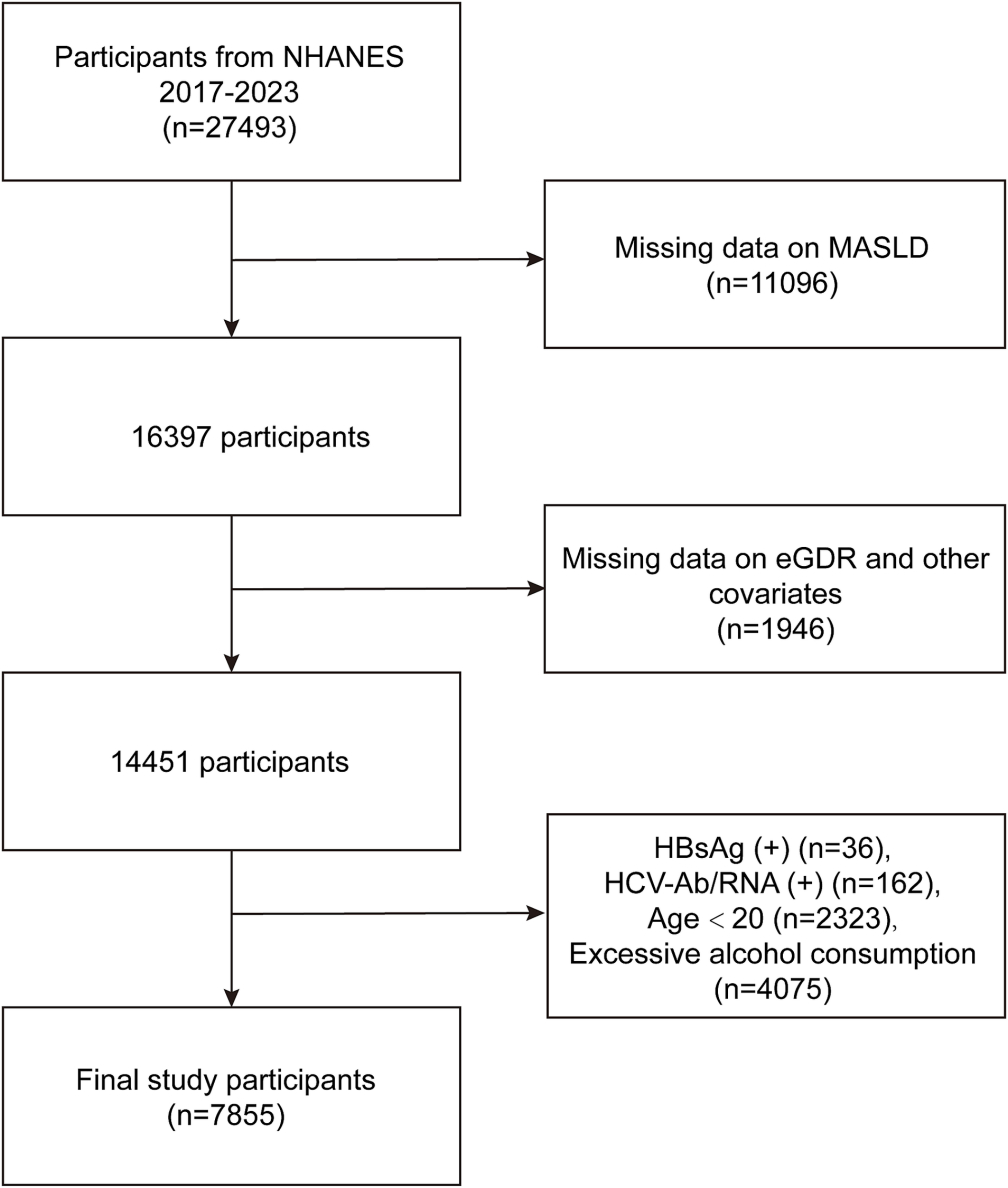
Flowchart of participant selection.

This study utilized data from the NHANES, a publicly available dataset collected by the National Center for Health Statistics. NHANES employs a complex, multistage probability sampling design to obtain a representative sample of the non-institutionalized US civilian population. Ethical approval for NHANES was granted by the Ethics Review Board of the National Center for Health Statistics. All NHANES participants provided written informed consent prior to data collection. Detailed information regarding NHANES study protocols, including ethical guidelines and informed consent procedures, is available on the NHANES website. The present analyses were conducted in accordance with the ethical principles outlined in the 1964 Declaration of Helsinki and its later amendments. In this study, we initiated with a total of 27,493 participants from the NHANES database (2017–2023). Participants were excluded due to missing data on MASLD (*n* = 11,096), lack of data on eGDR and other relevant covariates (*n* = 1,946), positive Hepatitis B surface antigen (HBsAg) status (*n* = 36), positive Hepatitis C antibodies or RNA (*n* = 162), age under 20 years (*n* = 2,323), and excessive alcohol consumption (*n* = 4,075), which was defined as >21 standard drinks per week for men and >14 standard drinks per week for women (27, 28) (Figure 1). Ultimately, a total of 7,855 participants were included for the analysis of the relationship between eGDR and MASLD.

### 2.2 Definition of eGDR

eGDR was determined using the following equation: 21.158 – [0.09 × waist circumference (WC)] − (3.407 × hypertension) − [0.551 × glycosylated hemoglobin (HbA1c)], where WC represents waist circumference (cm), Hypertension is a dichotomous variable (1 = presence of hypertension, 0 = absence of hypertension), and HbA1c is glycosylated hemoglobin (%) (29).

### 2.3 Definition of MASLD and liver fibrosis

The diagnosis of MASLD was based on established criteria requiring evidence of hepatic steatosis in conjunction with at least one of five metabolic risk factors (1). In this study, hepatic steatosis was assessed using the controlled attenuation parameter measured via vibration-controlled transient elastograph. The controlled attenuation parameter value of ≥ 268 dB/m was considered indicative of significant steatosis (30). To further investigate the association between eGDR and liver fibrosis, liver stiffness measurement values obtained from VCTE were also analyzed. Liver stiffness measurement provides a noninvasive and validated assessment of hepatic fibrosis and is expressed in kilopascals (kPa). Fibrosis stages were further categorized as follows: F2 (≥ 8.0 kPa), F3 (≥ 9.7 kPa), and F4 (≥ 13.7 kPa), corresponding to moderate, advanced, and severe fibrosis, respectively (28, 31).

### 2.4 Definition of AIP

The AIP was computed using the formula: log10 [triglyceride (TG) / high-density lipoprotein cholesterol (HDL-C)] (32, 33).

### 2.5 Covariates

Demographic variables included age, gender (male, female), race (non-Hispanic White, non-Hispanic Black, Mexican American, and other races), education level (categorized as less than high school, high school, and more than high school), and marital status (married/cohabiting, widowed/divorced/separated, and never married). Socioeconomic status was assessed using the poverty income ratio (PIR), categorized as <1, 1–3, and ≥3. Body mass index (BMI) was classified into three groups: <25, 25–30, and ≥30. Hypertension was defined as self-reported diagnosis by a healthcare provider, current use of antihypertensive medications, or average systolic blood pressure ≥ 140 mmHg or average diastolic blood pressure ≥ 90 mmHg. Diabetes was identified based on self-reported diagnosis, use of hypoglycemic medications, HbA1c ≥ 6.5% (measured using NGSP-aligned methods), or fasting blood glucose ≥ 7.0 mmol/L. Lifestyle factors included smoking status (defined as having smoked at least 100 cigarettes in a lifetime). Additionally, CVD was assessed through affirmative responses to questions regarding previous diagnoses of congestive heart failure, coronary heart disease, angina, heart attack, or stroke. A history of cancer was determined by self-reporting of any cancer or malignant tumor diagnosis. Clinical measures included WC (cm), total cholesterol (TC) (mmol/L), HDL-C (mmol/L), low-density lipoprotein cholesterol (LDL-C) (mmol/L), and TG (mmol/L). Finally, hyperlipidemia was defined as the presence of any of the following lipid abnormalities: HDL-C < 40 mg/dL in men or <50 mg/dL in women, LDL-C ≥ 130 mg/dL, TG ≥ 150 mg/dL, or TC ≥ 200 mg/dL (34). Statin use was assessed based on responses to relevant questionnaire items regarding current use of lipid-lowering medications.

### 2.6 Statistical analyses

All statistical analyses were carried out using R software (version 4.2) and EmpowerStats (v.4.1, http://www.empowerstats.com, X&Y Solutions, Inc., Boston, MA, United States), accounting for the complex sampling design of NHANES by incorporating appropriate sampling weights. Continuous variables were expressed as weighted mean ± standard deviation, while categorical variables were presented as frequencies and weighted proportions (%). Baseline characteristics across eGDR quartiles were compared using one-way ANOVA for normally distributed continuous variables, Kruskal-Wallis tests for non-normally distributed variables, and chi-square tests for categorical variables. Multivariable logistic regression models were constructed to evaluate the association between eGDR (as a continuous variable and in quartiles) and MASLD, as well as liver fibrosi. Three sequential models were developed: Model 1 (unadjusted), Model 2 (adjusted for age, gender, and race), and Model 3 (fully adjusted for age, gender, race, education level, marital status, PIR, BMI, smoking, diabetes, hypertension, HDL-C, LDL-C, TC, TG, CVD, cancer, hyperlipidemia, and statin use). To assess non-linear relationships, we employed generalized additive models (GAM) with smoothing splines, stratified by age, hypertension, diabetes, CVD and BMI. Threshold effects were examined using a two-piecewise linear regression model, and the optimal inflection point was identified. Subgroup analyses were conducted, and interaction terms were included in the regression models to assess effect modification by various factors. Mediation analysis was performed to quantify the proportion of the total effect of eGDR on MASLD mediated by the AIP. Sensitivity analysis was performed by excluding participants with extreme values of eGDR (eGDR <2 or ≥10) to test the robustness of the results. All statistical tests were two-tailed, and a *p*-value <0.05 was considered statistically significant.

## 3 Results

### 3.1 Participant characteristics

As shown in Table 1, this study included 7,855 participants from the NHANES 2017–2023 dataset, with an average age of 51.57 ± 17.31 years and a mean eGDR of 7.48 ± 2.80. The participants were categorized into four quartiles based on eGDR: Q1 (−2.84 to 4.97), Q2 (4.97 to 7.00), Q3 (7.00 to 9.53), and Q4 (9.53 to 12.88). Significant differences were observed across the quartiles in multiple demographic and clinical characteristics (all *p* < 0.0001), including age, gender, race, education level, marital status, PIR, BMI, smoking, diabetes, hypertension, CVD, cancer, hyperlipidemia, MASLD, liver fibrosis stage, WC, TC, HDL-C, LDL-C, and TG. Specifically, participants in higher eGDR quartiles tended to be younger, more likely to be female, have higher education levels and income, and lower BMI. The prevalence of diabetes, hypertension, hyperlipidemia, and CVD declined as eGDR increased. The proportion of smokers increased with higher eGDR, whereas the prevalence of MASLD markedly decreased across quartiles. A similar trend was observed for liver fibrosis, with advanced fibrosis (F2–F4) being most frequent in the lowest eGDR group and least frequent in the highest. Regarding metabolic parameters, participants in higher eGDR quartiles had lower WC, TG, and LDL-C, and higher HDL-C levels, suggesting a more favorable metabolic profile.

**Table 1:** Characteristics of study participants.

Characteristics^a^	eGDR				
	Q1	Q2	Q3	Q4	*p-*value
	−2.84-4.97	4.97–7.00	7.00–9.53	9.53–12.88	
Age (years)	59.15 ± 14.15	59.32 ± 15.40	49.90 ± 16.67	42.00 ± 16.03	<0.0001
Gender, *n* (%)					<0.0001
Male	1,032 (54.53%)	1,021 (53.84%)	1,051 (56.14%)	887 (48.71%)	
Female	930 (45.47%)	944 (46.16%)	913 (43.86%)	1,077 (51.29%)	
Race, *n* (%)					<0.0001
Mexican American	143 (5.47%)	157 (5.78%)	207 (7.65%)	146 (6.54%)	
Other Hispanic	176 (6.63%)	169 (7.08%)	187 (8.03%)	214 (9.37%)	
Non-Hispanic white	935 (65.27%)	871 (61.64%)	920 (63.42%)	841 (58.96%)	
Non-Hispanic black	529 (15.38%)	417 (12.65%)	316 (8.73%)	309 (8.9%)	
Other race	179 (7.25%)	351 (12.85%)	334 (12.18%)	454 (16.23%)	
Education level, *n* (%)					<0.0001
Less than high school	362 (12.11%)	361 (11.9%)	298 (9.62%)	244 (8.97%)	
High school	495 (30.02%)	450 (27.91%)	406 (24.71%)	377 (21.75%)	
More than high school	1,103 (57.87%)	1,149 (60.18%)	1,259 (65.67%)	1,341 (69.28%)	
Marital status, *n* (%)					<0.0001
Married or cohabitating	1,113 (63.95%)	1,170 (64.87%)	1,217 (65.99%)	1,184 (62.37%)	
Widowed, divorced, or separated	597 (24.83%)	573 (24.6%)	411 (17.1%)	294 (12.16%)	
Never married	251 (11.21%)	219 (10.53%)	331 (16.92%)	484 (25.47%)	
PIR, *n* (%)					<0.0001
<1	444 (19.18%)	393 (17.3%)	394 (16.74%)	404 (18.38%)	
1–3	726 (42.06%)	654 (37.06%)	673 (35.44%)	562 (30.89%)	
> = 3	529 (38.75%)	635 (45.64%)	670 (47.82%)	731 (50.73%)	
BMI (kg/m^2), *n* (%)					<0.0001
<25	25 (1.19%)	431 (20.52%)	376 (14.55%)	1,230 (61.94%)	
25–30	300 (13.35%)	932 (45.78%)	705 (36.59%)	670 (35.63%)	
> = 30	1,634 (85.46%)	592 (33.7%)	878 (48.85%)	60 (2.43%)	
Smoking, *n* (%)					<0.0001
No	878 (43.45%)	781 (37.47%)	688 (33.95%)	548 (27.69%)	
Yes	1,082 (56.55%)	1,183 (62.53%)	1,274 (66.05%)	1,415 (72.31%)	
Diabetes, *n* (%)					<0.0001
No	930 (50.83%)	1,517 (80.36%)	1719 (90.07%)	1917 (98.9%)	
Yes	1,032 (49.17%)	448 (19.64%)	245 (9.93%)	47 (1.1%)	
Hypertension, *n* (%)					<0.0001
No	55 (3.22%)	283 (17.54%)	1,629 (86.86%)	1964 (100.00%)	
Yes	1907 (96.78%)	1,682 (82.46%)	335 (13.14%)	0 (0.00%)	
CVD, *n* (%)					<0.0001
No	1,488 (79.43%)	1,645 (84.83%)	1800 (92.96%)	1898 (97.41%)	
Yes	474 (20.57%)	320 (15.17%)	163 (7.04%)	66 (2.59%)	
Cancer, *n* (%)					<0.0001
No	344 (17.98%)	342 (17.37%)	246 (10.85%)	154 (7.8%)	
Yes	1,617 (82.02%)	1,622 (82.63%)	1717 (89.15%)	1810 (92.2%)	
Hyperlipidemia, *n* (%)					<0.0001
No	672 (33.54%)	762 (39.44%)	731 (38.05%)	981 (51.65%)	
Yes	1,214 (66.46%)	1,133 (60.56%)	1,179 (61.95%)	931 (48.35%)	
MASLD, *n* (%)					<0.0001
No	519 (21.58%)	1,098 (51.11%)	1,079 (53.82%)	1719 (87.77%)	
Yes	1,443 (78.42%)	867 (48.89%)	885 (46.18%)	245 (12.23%)	
	Q1	Q2	Q3	Q4	*p*-value
Liver fibrosis, *n* (%)					<0.0001
F2	153 (7.99%)	102 (4.66%)	87 (4.86%)	34 (1.73%)	
F3	190 (8.76%)	60 (2.97%)	42 (1.83%)	20 (1.26%)	
F4	190 (11.47%)	41 (1.82%)	37 (2.07%)	13 (0.70%)	
WC (cm)	119.46 ± 12.92	101.97 ± 13.25	102.16 ± 11.44	84.82 ± 7.67	<0.0001
TC (mmol/L)	4.67 ± 1.08	4.91 ± 1.09	4.98 ± 1.05	4.81 ± 1.08	<0.0001
HDL-C (mmol/L)	1.22 ± 0.31	1.37 ± 0.35	1.32 ± 0.35	1.47 ± 0.37	<0.0001
LDL-C (mmol/L)	2.66 ± 0.96	2.93 ± 0.88	3.04 ± 0.88	2.84 ± 1.02	<0.0001
TG (mmol/L)	2.00 ± 1.33	1.66 ± 1.03	1.65 ± 1.42	1.18 ± 0.71	<0.0001

a Data are presented as frequencies (%) or mean ± standard deviation.

Q1, Quartile 1; Q2, Quartile 2; Q3, Quartile 3; Q4, Quartile 4; eGDR, estimated glucose disposal rate; MASLD, metabolic dysfunction-associated steatotic liver disease; PIR, poverty to income ratio; BMI, body mass index; CVD, cardiovascular disease; WC, waist circumference; TC, total cholesterol; HDL-C, high-density lipoprotein cholesterol; LDL-C, low-density lipoprotein cholesterol; TG, triglyceride.

### 3.2 Associations between eGDR and MASLD and liver fibrosis

The multivariate logistic regression analyses examined the associations between eGDR and MASLD, as well as liver fibrosis (Table 2). In the unadjusted model (Model 1), each unit increase in continuous eGDR was significantly associated with lower odds of MASLD (OR = 0.70, 95% CI: 0.68–0.71, *p* < 0.0001). In the fully adjusted model (Model 3), which accounted for age, gender, race, education level, marital status, PIR, BMI, smoking, diabetes, hypertension, HDL-C, LDL-C, TC, TG, CVD, cancer, hyperlipidemia, and statin use, the association remained significant (OR = 0.62, 95% CI: 0.53–0.72, *p* < 0.0001). When eGDR was analyzed as quartiles, participants in Q2, Q3, and Q4 had progressively lower odds of MASLD compared to those in Q1. In Model 1, the odds ratios for Q2, Q3, and Q4 were 0.28 (95% CI: 0.25–0.32), 0.30 (95% CI: 0.26–0.34), and 0.05 (95% CI: 0.04–0.06), respectively (all *p* < 0.0001). In Model 3, the odds ratios were 0.56 (95% CI: 0.37–0.84, *p* = 0.0047), 0.25 (95% CI: 0.12–0.50, *p* = 0.0001), and 0.13 (95% CI: 0.05–0.31, *p* < 0.0001), respectively. These findings indicate a significant inverse relationship between eGDR and MASLD, with higher eGDR associated with reduced odds of MASLD.

**Table 2:** Associations between eGDR and MASLD and liver fibrosis.

Variable	Model 1	*p*-value	Model 2	*p*-value	Model 3	*p*-value
MASLD						
eGDR continuous	0.70 (0.68, 0.71)	<0.0001	0.65 (0.64, 0.67)	<0.0001	0.62 (0.53, 0.72)	<0.0001
eGDR quartile						
Q1	Reference		Reference		Reference	
Q2	0.28 (0.25, 0.32)	<0.0001	0.26 (0.23, 0.30)	<0.0001	0.56 (0.37, 0.84)	0.0047
Q3	0.30 (0.26, 0.34)	<0.0001	0.23 (0.20, 0.26)	<0.0001	0.25 (0.12, 0.50)	0.0001
Q4	0.05 (0.04, 0.06)	<0.0001	0.03 (0.03, 0.04)	<0.0001	0.13 (0.05, 0.31)	<0.0001
Liver fibrosis						
eGDR continuous	0.71 (0.69, 0.73)	<0.0001	0.71 (0.69, 0.73)	<0.0001	0.50 (0.42, 0.58)	<0.0001
eGDR quartile						
Q1	Reference		Reference		Reference	
Q2	0.31 (0.26, 0.37)	<0.0001	0.31 (0.26, 0.37)	<0.0001	0.24 (0.13, 0.44)	<0.0001
Q3	0.25 (0.21, 0.30)	<0.0001	0.24 (0.20, 0.29)	<0.0001	0.06 (0.02, 0.16)	<0.0001
Q4	0.09 (0.07, 0.12)	<0.0001	0.09 (0.07, 0.12)	<0.0001	0.05 (0.01, 0.19)	<0.0001

Model 1 was adjusted for none. Model 2 was adjusted for age, gender, and race. Model 3 was adjusted for age, gender, race, education level, marital status, PIR, BMI, smoking, diabetes, hypertension, HDL-C, LDL-C, TC, TG, CVD, cancer, hyperlipidemia, and statin use. Q1, Quartile 1; Q2, Quartile 2; Q3, Quartile 3; Q4, Quartile 4; eGDR, estimated glucose disposal rate; MASLD, metabolic dysfunction-associated steatotic liver disease; PIR, poverty to income ratio; BMI, body mass index; CVD, cardiovascular disease; TC, total cholesterol; HDL-C, high-density lipoprotein cholesterol; LDL-C, low-density lipoprotein cholesterol; TG, triglyceride.

Similarly, higher eGDR was also significantly associated with lower odds of liver fibrosis. In Model 1, each unit increase in eGDR was associated with an OR of 0.71 (95% CI: 0.69–0.73, *p* < 0.0001), and this relationship persisted in Model 3 (OR = 0.50, 95% CI: 0.42–0.58, *p* < 0.0001). Compared with Q1, the odds ratios for liver fibrosis in Q2, Q3, and Q4 were 0.31 (95% CI: 0.26–0.37), 0.25 (95% CI: 0.21–0.30), and 0.09 (95% CI: 0.07–0.12), respectively, in the unadjusted model (all *p* < 0.0001). After full adjustment in Model 3, the associations remained significant, with ORs of 0.24 (95% CI: 0.13–0.44), 0.06 (95% CI: 0.02–0.16), and 0.05 (95% CI: 0.01–0.19) for Q2, Q3, and Q4, respectively (all *p* < 0.0001). These results suggest that higher eGDR is strongly and independently associated with a lower risk of liver fibrosis.

### 3.3 Associations of eGDR with MASLD and liver fibrosis across stratified populations

**Figure 2:**
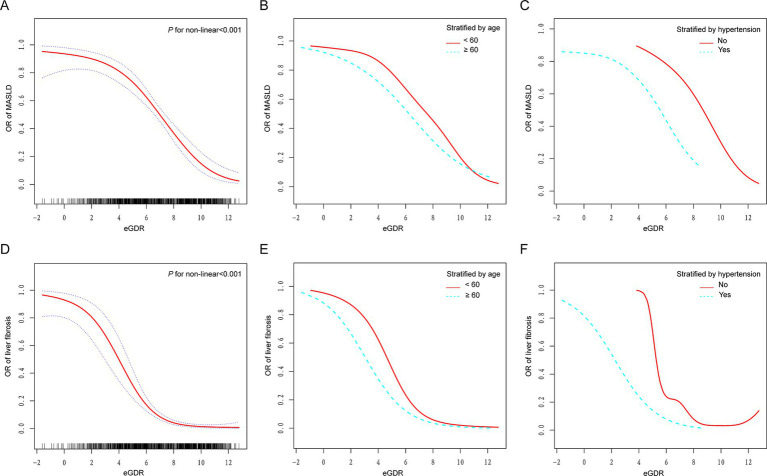
Dose–response relationships between eGDR and MASLD and liver fibrosis. **(A,D)** A non-linear association was found between eGDR and both MASLD and liver fibrosis (*P* for non-linearity <0.001). **(B,E)** Stratified by age (60 years). **(C,F)** Stratified by hypertension. Adjusted for age, gender, race, education level, marital status, PIR, BMI, smoking, diabetes, hypertension, HDL-C, LDL-C, TC, TG, CVD, cancer, hyperlipidemia, and statin use. eGDR, estimated glucose disposal rate; MASLD, metabolic dysfunction-associated steatotic liver disease; PIR, poverty to income ratio; BMI, body mass index; CVD, cardiovascular disease; TC, total cholesterol; HDL-C, high-density lipoprotein cholesterol; LDL-C, low-density lipoprotein cholesterol; TG, triglyceride.

Using generalized additive models (GAMs) and adjusting for all covariates, a significant non-linear association was observed between eGDR and both MASLD and liver fibrosis (*P* for non-linearity <0.001; Figures 2A,D). In subgroup analyses stratified by age (Figures 2B,E) and hypertension status (Figures 2C,F), the dose–response curves showed evident leftward shifts among participants aged ≥60 years or those with hypertension, compared to their counterparts aged <60 years or without hypertension. Additional stratified analyses by diabetes (Supplementary Figures S1A,D), CVD (Supplementary Figures S1B,E), and BMI categories (Supplementary Figures S1C,F) revealed distinct dose–response relationships across subgroups, further indicating that the association between eGDR and both MASLD and liver fibrosis varies by metabolic and clinical status.

### 3.4 Threshold effect analysis of the relationship between eGDR and MASLD

The threshold effect analysis evaluates the relationship between eGDR and MASLD (Table 3). Model II identified an inflection point (K) at 3.25 for eGDR. When eGDR was less than the inflection point (K < 3.25), no significant association with MASLD was found (OR = 0.94, 95% CI: 0.64–1.37, *p* = 0.7333). However, when eGDR exceeded the inflection point (K > 3.25), a significant inverse association with MASLD was observed (OR = 0.56, 95% CI: 0.48–0.67, *p* < 0.0001). The *p*-value for the log-likelihood ratio test was 0.039, indicating the presence of a threshold effect. These results suggest a non-linear association between eGDR and MASLD, with markedly lower odds of MASLD observed among individuals with eGDR greater than 3.25.

**Table 3:** Threshold effect analysis of the relationship between eGDR and MASLD.

Outcome	MASLD	*p*-value
	OR (95%CI)	
Model I		
One line effect	0.62 (0.53, 0.72)	<0.0001
Model II		
Inflection point (K)	3.25	
<K	0.94 (0.64, 1.37)	0.7333
>K	0.56 (0.48, 0.67)	<0.0001
*P* for log-likelihood ratio test		0.039

Adjusted for age, gender, race, education level, marital status, PIR, BMI, smoking, diabetes, hypertension, HDL-C, LDL-C, TC, TG, CVD, cancer, hyperlipidemia, and statin use. eGDR, estimated glucose disposal rate; MASLD, metabolic dysfunction-associated steatotic liver disease; PIR, poverty to income ratio; BMI, body mass index; CVD, cardiovascular disease; TC, total cholesterol; HDL-C, high-density lipoprotein cholesterol; LDL-C, low-density lipoprotein cholesterol; TG, triglyceride.

### 3.5 Subgroup analysis

**Figure 3:**
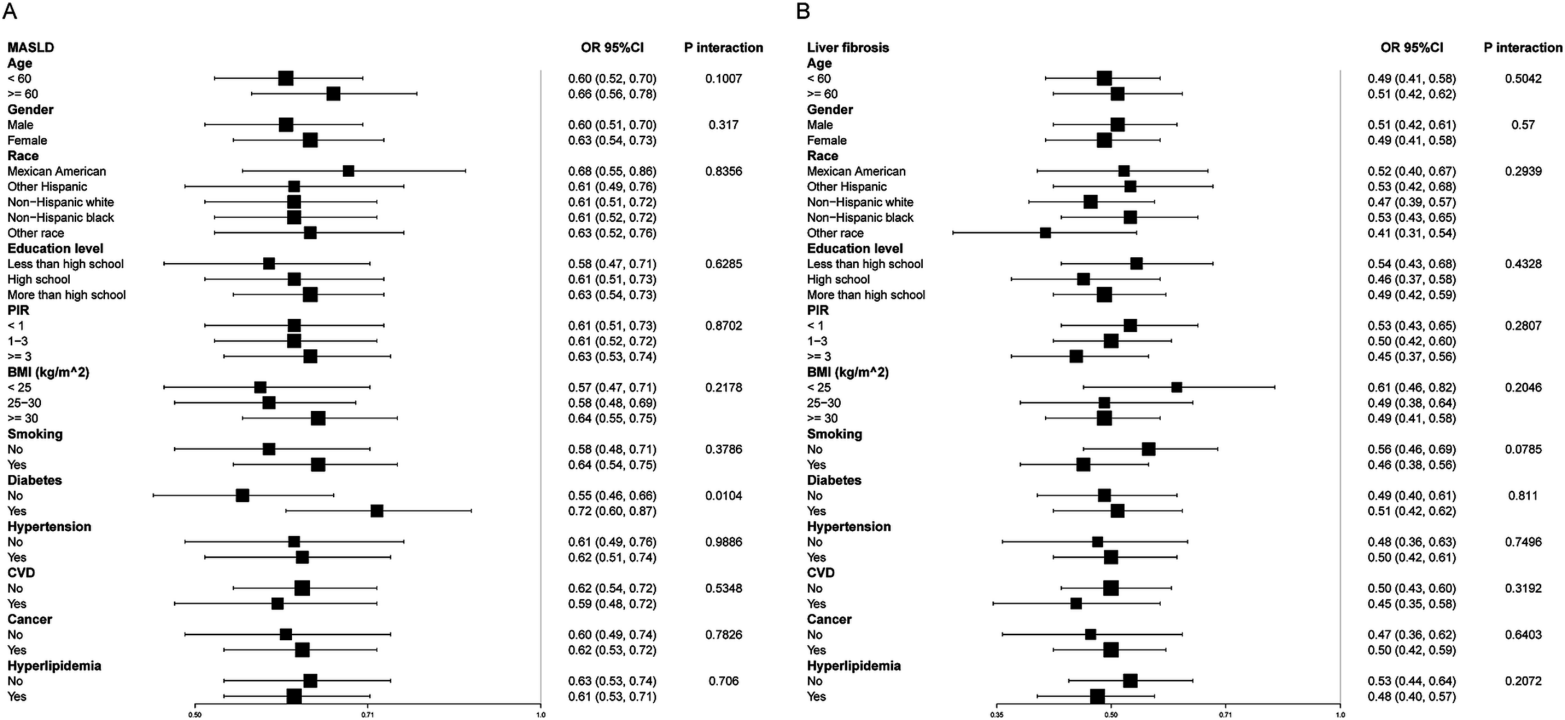
Subgroup analysis of eGDR and MASLD **(A)** and liver fibrosis **(B)**. Adjusted for age, gender, race, education level, marital status, PIR, BMI, smoking, diabetes, hypertension, HDL-C, LDL-C, TC, TG, CVD, cancer, hyperlipidemia, and statin use. eGDR, estimated glucose disposal rate; MASLD, metabolic dysfunction-associated steatotic liver disease; PIR, poverty to income ratio; BMI, body mass index; CVD, cardiovascular disease; TC, total cholesterol; HDL-C, high-density lipoprotein cholesterol; LDL-C, low-density lipoprotein cholesterol; TG, triglyceride.

Subgroup analyses were conducted to evaluate the association between eGDR and MASLD across various demographic and clinical strata (Figure 3A). A significant interaction was observed for diabetes status (*P*-interaction = 0.0104), while no significant interactions were found for age, gender, race, education level, PIR, BMI, smoking status, hypertension, CVD, cancer, or hyperlipidemia. Specifically, the inverse association between eGDR and MASLD was more pronounced in participants without diabetes (OR = 0.55, 95% CI: 0.46–0.66) compared to those with diabetes (OR = 0.72, 95% CI: 0.60–0.87). Among participants aged <60 years and ≥60 years, the associations were similar (OR = 0.60, 95% CI: 0.52–0.70 vs. OR = 0.66, 95% CI: 0.56–0.78), with no significant interaction detected (*P*-interaction = 0.1007). Similarly, subgroup analyses for liver fibrosis (Figure 3B) showed consistent inverse associations between eGDR and liver fibrosis across all strata. However, none of the interaction terms reached statistical significance, suggesting that the relationship between eGDR and liver fibrosis was generally robust and not significantly modified by demographic or clinical characteristics.

### 3.6 Mediation analysis

**Figure 4:**
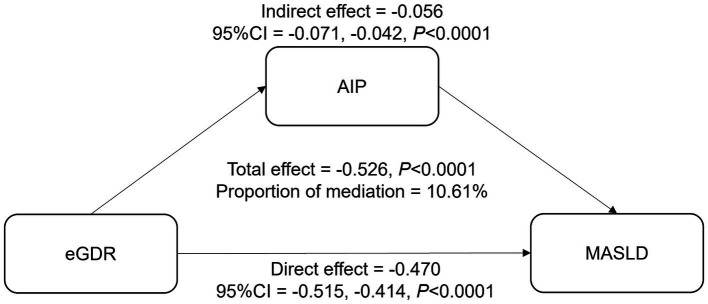
The mediation effect of AIP between eGDR and MASLD. Adjusted for age, gender, race, education level, marital status, PIR, BMI, smoking, diabetes, hypertension, CVD, and cancer. eGDR, estimated glucose disposal rate; MASLD, metabolic dysfunction-associated steatotic liver disease; AIP, atherogenic index of plasma; PIR, poverty to income ratio; BMI, body mass index; CVD, cardiovascular disease.

The mediation analysis examines the role of AIP in the relationship between eGDR and MASLD (Table 4; Figure 4). The total effect of eGDR on MASLD was significant, with a coefficient of −0.526 (95% CI: −0.566, −0.475, *p* < 0.0001). AIP was found to mediate part of this association, with an indirect effect of −0.056 (95% CI: −0.071, −0.042, *p* < 0.0001). The direct effect of eGDR on MASLD, after accounting for AIP, was −0.470 (95% CI: −0.515, −0.414, *p* < 0.0001). The proportion of the total effect mediated by AIP was 10.61%. These findings suggest that AIP partially mediates the relationship between eGDR and MASLD, highlighting its potential role in this association.

**Table 4:** Mediation analysis of AIP in the association between eGDR and MASLD.

Independent variable	Mediator	Total effect	*p*-value	Indirect effect	*p*-value	Direct effect	*p*-value	Proportion mediated (%)
		Coefficient (95% CI)		Coefficient (95% CI)		Coefficient (95% CI)		
eGDR	AIP	−0.526 (−0.566, −0.475)	<0.0001	−0.056 (−0.071, −0.042)	<0.0001	−0.470 (−0.515, −0.414)	<0.0001	10.61%

Adjusted for age, gender, race, education level, marital status, PIR, BMI, smoking, diabetes, hypertension, CVD, and Cancer. eGDR, estimated glucose disposal rate; MASLD, metabolic dysfunction-associated steatotic liver disease; AIP, atherogenic index of plasma; PIR, poverty to income ratio; BMI, body mass index; CVD, cardiovascular disease.

### 3.7 Sensitivity analysis

To verify robustness, a sensitivity analysis excluding individuals with eGDR <2 or ≥10 was performed (Supplementary Table S1). The inverse association between eGDR and both MASLD and liver fibrosis remained consistent and significant. For MASLD, eGDR (continuous) was significantly associated in all models, with ORs ranging from 0.77 to 0.60 (all *p* < 0.0001). Quartile analyses showed a dose–response trend, with the lowest odds in Q4 (OR = 0.17, 95% CI: 0.06–0.44, *p* = 0.0003 in Model 3). Similarly, for liver fibrosis, eGDR was inversely associated across models (OR = 0.46 in Model 3, 95% CI: 0.37–0.57). Higher quartiles of eGDR were linked to progressively lower odds, with Q4 showing a strong association (OR = 0.06, 95% CI: 0.01–0.28, *p* = 0.0005).

## 4 Discussion

In this study, we found a significant inverse association between eGDR and both MASLD and liver fibrosis, which remained robust after adjusting for multiple confounders. These associations exhibited clear non-linear patterns, with a more pronounced inverse relationship observed when eGDR exceeded a threshold value of 3.25 for MASLD. Subgroup analyses revealed that the inverse relationship between eGDR and MASLD was more pronounced in individuals without diabetes. Smoothing curve fitting revealed significant leftward shifts in the dose–response curves among individuals aged ≥60 years or with hypertension, indicating that the relationships between eGDR and both MASLD and liver fibrosis differ across metabolic and clinical subgroups. Additionally, mediation analysis indicated that AIP partially mediated the association between eGDR and MASLD, accounting for approximately 10.6% of the total effect, highlighting its potential role in this pathway.

The eGDR, initially developed to assess IR in type 1 diabetes, has been validated as a reliable surrogate marker of IR through direct comparison with hyperinsulinemic-euglycemic clamp-measured glucose disposal rates in both type 1 diabetes and type 2 diabetes populations (21, 35, 36). Notably, eGDR exhibits superior predictive performance in CVD risk stratification compared to other IR indices, such as the TyG, homeostasis model assessment of IR, quantitative insulin sensitivity check index, and metabolic score for IR (37). These advantages position eGDR as a clinically practical and comprehensive tool for assessing IR-related metabolic derangements. In a 2025 NHANES study involving 10,690 participants with diabetes or prediabetes, Liao et al. demonstrated that lower eGDR independently predicted elevated risks of CVD, including coronary artery disease, heart failure, and stroke (37). Longitudinal studies further linked reduced eGDR to diabetic nephropathy progression and increased all-cause mortality in type 2 diabetes (36), with broader applicability confirmed by its association with cardiovascular and all-cause mortality in populations regardless of diabetes status (38).

In recent years, research on the relationship between eGDR and liver diseases, particularly NAFLD and MASLD, has gained attention. However, studies specifically examining eGDR in this context remain limited. In 2022, Marieke et al. (24) observed that lower eGDR was significantly associated with the presence of NAFLD in patients with type 1 diabetes. Similarly, Agata et al. (19) in 2023 reported a comparable association, demonstrating that reduced eGDR independently correlated with an elevated risk of NAFLD in the same patient population. The large-scale longitudinal evidence further supports the predictive utility of eGDR in MASLD risk assessment. The study by Liu et al. (39) similarly found an inverse association between eGDR and both MASLD and liver fibrosis. However, our study offers several key advantages and additional insights. First, we utilized the most recent NHANES data (2017–2023), which allowed us to work with a sample size more than twice as large as the study by Liu et al. This larger sample size enhances the robustness and generalizability of our findings. Second, our study incorporated a more comprehensive set of confounders in the adjusted analyses, allowing for a more nuanced exploration of the factors that influence the relationship between eGDR and liver disease. Third, we conducted subgroup analyses to examine the dose–response relationships across different population groups. Furthermore, our study applied threshold effect analysis, identifying a critical inflection point (eGDR > 3.25) beyond which the relationship with MASLD became more pronounced. In addition, we employed mediation analysis, which revealed that AIP partially mediated the relationship between eGDR and MASLD, providing deeper insights into the underlying mechanisms.

Subgroup analyses in our study revealed that the inverse association between eGDR and MASLD was more pronounced in those without diabetes. The absence of diabetes likely indicates a lower baseline level of IR, which enhances the beneficial effects of improved insulin sensitivity on liver health. The non-diabetic subgroup may therefore exhibit more significant improvements in metabolic health when eGDR is elevated, resulting in a more pronounced reduction in MASLD risk. Furthermore, our stratified analysis using smoothing curve fitting revealed that, compared to younger individuals and those without hypertension, older individuals and those with hypertension experienced a greater reduction in MASLD risk at equivalent higher levels of eGDR. This suggests that improving insulin sensitivity confers even more substantial benefits to high-risk populations. The observed effect may reflect the greater metabolic dysregulation and IR present in these individuals, where improved insulin sensitivity may be linked to a lower likelihood of MASLD development. These findings emphasize the need for tailored strategies in managing MASLD risk, suggesting that interventions aimed at improving insulin sensitivity, such as lifestyle modifications or pharmacologic therapies, could yield greater benefits for high-risk groups, including older adults and those with hypertension. Future studies should further explore whether these strategies improve long-term clinical outcomes in these populations and investigate the underlying mechanisms driving the differential responses to eGDR in various subgroups.

Our mediation analysis suggests that the AIP may partially mediate the relationship between eGDR and MASLD. AIP reflects the balance between pro-atherogenic and anti-atherogenic processes, playing a crucial role in lipid metabolism and IR (40, 41). Previous studies have shown that elevated AIP increases IR risk and disrupts lipid metabolism (42, 43), which may explain its involvement in MASLD development. Furthermore, Li et al. (44) have demonstrated a positive correlation between AIP and MASLD, suggesting AIP as a potential predictor for the disease. Our findings, showing that AIP mediates about 10.61% of the effect between eGDR and MASLD, support the hypothesis that AIP may play a role in this association. These results highlight AIP as a potential target for MASLD management in insulin-resistant populations.

The exact mechanisms underlying the observed relationship between eGDR and both MASLD and liver fibrosis remain incompletely understood, but several plausible pathways can be hypothesized based on existing literature. eGDR, a reliable indicator of IR, reflects the combined effects of WC, hypertension, and HbA1c, all of which independently and synergistically contribute to the pathogenesis of MASLD. IR exacerbates adipose tissue lipolysis, increasing free fatty acid (FFA) flux to the liver and overwhelming hepatic lipid export capacity, a hallmark of MASLD pathogenesis (19, 45). An increase in WC signifies obesity, which is often associated with the accumulation of visceral fat. This fat accumulation can produce inflammatory mediators such as tumor necrosis factor-alpha (TNF-*α*) and interleukin-6 (IL-6) (46, 47). These mediators may further induce low-grade systemic inflammation, which contributes to IR and subsequently promotes the development of MASLD (48). Similarly, hypertension exacerbates endothelial dysfunction and further impairs insulin sensitivity (49), while elevated HbA1c reflects poor glycemic control, a key factor in the progression of liver damage (50, 51). Together, these factors create a vicious cycle of IR, lipid accumulation, and liver damage, ultimately leading to the development and progression of both MASLD and liver fibrosis.

This study has several strengths, including the utilization of the latest NHANES data (2017–2023) and adherence to the MASLD diagnostic criteria. The application of GAM with smoothing splines provided robust visualization of the non-linear dose–response relationship between eGDR and MASLD, while threshold effect analysis identified a critical inflection point (K = 3.25 mg/kg/min) that may inform clinical intervention targets. Subgroup and mediation analyses further delineated population-specific risk gradients and mechanistic pathways. In terms of clinical implications, this study suggests that eGDR, a marker of insulin sensitivity, is inversely associated with both MASLD and liver fibrosis, highlighting its potential as a biomarker for early detection and risk stratification. Derived from routine clinical data like fasting glucose, BMI, and blood pressure, eGDR offers a simple, cost-effective tool for screening, particularly in primary care where liver disease is often underdiagnosed. Its main advantage is identifying individuals at higher risk before clinical symptoms appear, enabling earlier intervention. However, challenges include its applicability to diverse populations and the need for further validation. Additionally, eGDR may not fully capture liver disease complexity, and combining it with other biomarkers or imaging techniques could provide a more comprehensive assessment. In conclusion, while promising, further research is needed to refine eGDR’s clinical utility and role in disease management.

However, there are also some limitations. The cross-sectional design precludes causal inference, and reliance on self-reported covariates may introduce measurement bias. Despite employing rigorous exclusion criteria, residual confounding from unmeasured factors cannot be excluded — for instance, physical activity, an important covariate that may influence the association between eGDR and MASLD, was not accounted for in the analysis. Furthermore, the lack of longitudinal mortality data in recent NHANES cycles limits prognostic interpretation. Moreover, generalizability to non-U.S. populations requires validation, particularly in regions with distinct metabolic risk profiles. While several baseline characteristics demonstrated statistically significant differences across eGDR quartiles, the actual effect sizes were small. This may reflect the influence of large sample size rather than true clinical significance. Another limitation is that eGDR was assessed only at baseline, which may not fully capture its dynamic relationship with MASLD. Longitudinal studies with repeated measurements are needed to assess temporal and causal associations. Future studies should include longitudinal data to establish causal relationships and explore potential therapeutic interventions targeting eGDR and AIP. Additionally, more prospective research is needed to further validate the relationship between eGDR and MASLD.

## 5 Conclusion

In this study, we identified a significant inverse association between eGDR and both MASLD and liver fibrosis. These associations showed non-linear patterns, with a more pronounced inverse relationship observed when eGDR exceeded 3.25. Subgroup analyses indicated that the inverse association between eGDR and MASLD was more evident in individuals without diabetes. Moreover, smoothing curve fitting revealed that the dose–response relationships between eGDR and both MASLD and liver fibrosis varied by metabolic and clinical status, as evidenced by significant leftward shifts among individuals aged ≥60 years or with hypertension. Finally, mediation analysis suggested that the AIP partially mediated the association between eGDR and MASLD.

## Data Availability

The datasets presented in this study can be found in online repositories. The names of the repository/repositories and accession number(s) can be found below: https://wwwn.cdc.gov/nchs/nhanes/default.aspx.
