# Supplementary material for: Association between eGDR and MASLD and liver fibrosis: a cross-sectional study based on NHANES 2017–2023
Source: Front Med (Lausanne). 2025 May 30;12:1579879. doi: 10.3389/fmed.2025.1579879 (PMC12162473; doi:10.3389/fmed.2025.1579879)
Supplement: Supplementary file 1 [file Data_Sheet_1.docx]

Supplementary Material

# Supplementary Figures and Tables

Table S1. Sensitivity Analysis of eGDR and MASLD/Liver Fibrosis After Excluding Participants with eGDR <2 or ≥10

|  | Model 1 | *P*-value | Model 2 | *P*-value | Model 3 | *P*-value |
| --- | --- | --- | --- | --- | --- | --- |
| MASLD |  |  |  |  |  |  |
| eGDR continuous | 0.77 (0.75, 0.79) | <0.0001 | 0.72 (0.70, 0.74) | <0.0001 | 0.60 (0.51, 0.71) | <0.0001 |
| eGDR quartile |  |  |  |  |  |  |
| Q1 | Reference |  | Reference |  | Reference |  |
| Q2 | 0.32 (0.28, 0.36) | <0.0001 | 0.29 (0.25, 0.33) | <0.0001 | 0.56 (0.37, 0.85) | 0.0065 |
| Q3 | 0.33 (0.29, 0.38) | <0.0001 | 0.25 (0.21, 0.28) | <0.0001 | 0.24 (0.12, 0.50) | 0.0001 |
| Q4 | 0.15 (0.12, 0.18) | <0.0001 | 0.10 (0.08, 0.12) | <0.0001 | 0.17 (0.06, 0.44) | 0.0003 |
| Liver fibrosis |  |  |  |  |  |  |
| eGDR continuous | 0.72 (0.70, 0.75) | <0.0001 | 0.71 (0.69, 0.74) | <0.0001 | 0.46 (0.37, 0.57) | <0.0001 |
| eGDR quartile |  |  |  |  |  |  |
| Q1 | Reference |  | Reference |  | Reference |  |
| Q2 | 0.38 (0.31, 0.45) | <0.0001 | 0.37 (0.31, 0.45) | <0.0001 | 0.27 (0.14, 0.49) | <0.0001 |
| Q3 | 0.30 (0.25, 0.37) | <0.0001 | 0.29 (0.24, 0.36) | <0.0001 | 0.06 (0.02, 0.16) | <0.0001 |
| Q4 | 0.11 (0.06, 0.18) | <0.0001 | 0.10 (0.06, 0.17) | <0.0001 | 0.06 (0.01, 0.28) | 0.0005 |

Model 1 was adjusted for none. Model 2 was adjusted for age, gender, and race. Model 3 was adjusted for age, gender, race, education level, marital status, PIR, BMI, smoking, diabetes, hypertension, HDL-C, LDL-C, TC, TG, CVD, cancer, hyperlipidemia, and statin use. Abbreviations: Q1, Quartile 1; Q2, Quartile 2; Q3, Quartile 3; Q4, Quartile 4; eGDR, estimated glucose disposal rate; MASLD, metabolic dysfunction-associated steatotic liver disease; PIR, poverty to income ratio; BMI, body mass index; CVD, cardiovascular disease; TC, total cholesterol‌; HDL-C, high-density lipoprotein cholesterol; LDL-C, low-density lipoprotein cholesterol; TG, triglyceride.

Figure S1. Dose-response relationships between eGDR and MASLD and liver fibrosis

(A, D) Stratified by diabetes. (B, E) Stratified by CVD. (C, F) Stratified by BMI. Adjusted for age, gender, race, education level, marital status, PIR, BMI, smoking, diabetes, hypertension, HDL-C, LDL-C, TC, TG, CVD, cancer, hyperlipidemia, and statin use. Abbreviations: eGDR, estimated glucose disposal rate; MASLD, metabolic dysfunction-associated steatotic liver disease; PIR, poverty to income ratio; BMI, body mass index; CVD, cardiovascular disease; TC, total cholesterol‌; HDL-C, high-density lipoprotein cholesterol; LDL-C, low-density lipoprotein cholesterol; TG, triglyceride.
