# Supplementary figures and images for: Association between eGDR and MASLD and liver fibrosis: a cross-sectional study based on NHANES 2017–2023
Source: Front Med (Lausanne). 2025 May 30;12:1579879. doi: 10.3389/fmed.2025.1579879 (PMC12162473; doi:10.3389/fmed.2025.1579879)

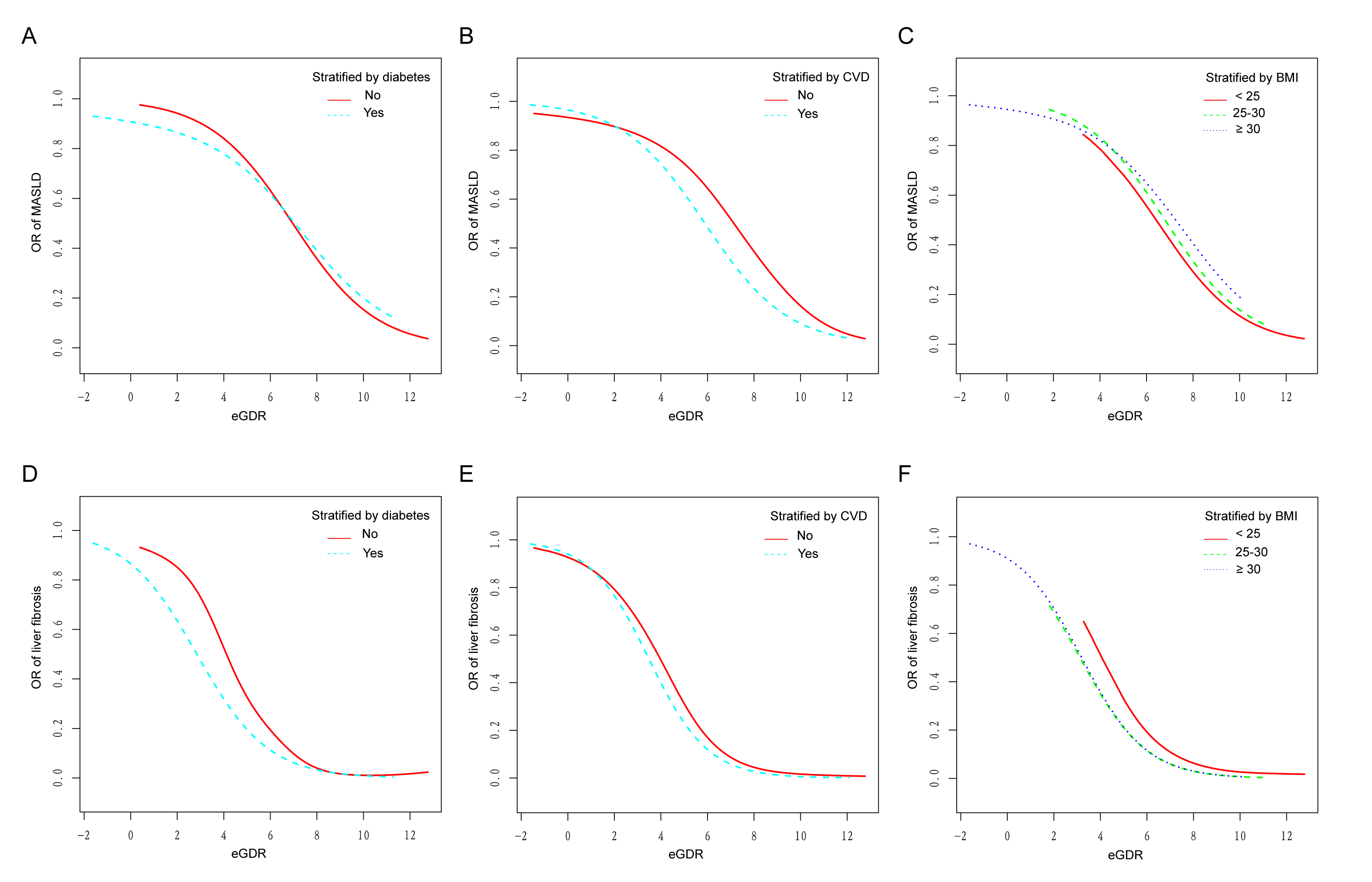

Supplement: Supplementary file 2 [file Figure_1.TIF]
